# Cardiopulmonary Resonance Function and Indices—A Quantitative Measurement for Respiratory Sinus Arrhythmia

**DOI:** 10.3389/fphys.2020.00867

**Published:** 2020-08-05

**Authors:** Jiajia Cui, Zhipei Huang, Jiankang Wu, Hong Jiang

**Affiliations:** ^1^Sensor Networks and Application Research Center, School of Electronic, Electrical and Communication Engineering, University of Chinese Academy of Sciences, Beijing, China; ^2^CAS Institute of Healthcare Technologies, Nanjing, China; ^3^Department of Cardiology, Integrated Chinese and Western Medicine, China-Japan Friendship Hospital, Beijing, China

**Keywords:** heart rate variability, respiratory sinus arrhythmia, spectral G-causality, cardiopulmonary interaction, coupled resonance

## Abstract

Respiratory sinus arrhythmia (RSA) represents a physiological phenomenon of cardiopulmonary interaction. It is known as a measure of efficiency of the circulation system, as well as a biomarker of cardiac vagal and well-being. In this article, RSA is modeled as modulation of heart rate by respiration in an interactive cardiopulmonary system with the most effective system state of resonance. By mathematically modeling of this modulation, we propose a quantitative measurement for RSA referred to as “Cardiopulmonary Resonance Function (CRF) and Cardiopulmonary Resonance Indices (CRI),” which are derived by disentanglement of the RR-intervals series into respiratory-modulation component, R-HRV, and the rest, NR-HRV using spectral G-causality. Evaluation of CRI performance in quantifying RSA has been conducted in the scenarios of paced breathing and in the different sleep stages. The preliminary experimental results have shown superior representation ability of CRF and CRI compared to Heart Rate Variability (HRV) and Cardiopulmonary Coupling index (CPC).

## Introduction

There is an urgent need for quantitative assessment of autonomic nervous function. Heart rate variability (HRV) is widely used as a non-invasive method. Particularly, low-frequency (LF, 0.04–0.15 Hz) and high-frequency (HF, 0.15–0.4 Hz) spectral components of HRV are used as the separate metrics of sympathetic and vagal(parasympathetic) functions (Appel et al., 1989). But as a simplified framework, HRV lacks solid physiological foundation, is not able to accommodate varieties of clinical cases (Hayano and Yuda, 2019). For example, HRV measures will change significantly in different physiologic states such as wake and sleep, exercise and rest, circadian rhythms, as well as with pathologic conditions (Task Force of the European Society of Cardiology the North American Society of Pacing Electrophysiology, 1996).

Cardiopulmonary interaction plays important role in the circulation system, and physiologically presents as respiratory sinus arrhythmia (RSA) phenomenon. RSA is regarded as a non-invasive measure of parasympathetic cardiac control (Katona and Jih, 1975; Topcu et al., 2018). The vagal origin of RSA can be found in the vagal synapses, which are faster than the sympathetic ones and are therefore able to translate central respiratory oscillations present in the brainstem to changes of cardiac sinus node discharge rate, which is not capable for slow sympathetic synapses. Tracking the autonomic regulation in RSA using the electrocardiogram and respiratory measurements is a feasible and important approach to gain our knowledge toward autonomic nervous system and its clinical applications.

The quantitative study of RSA has profound significance in physiology and pathology, as well as extensive clinical applications. Some studies have shown that RSA reaches a relatively stable state in deep sleep (Bernston et al., 1997) and a study of the hibernation of 37 polar bears in the University of Minnesota found that RSA reached their peak during the hibernation. At the same time, as a vagal inflammatory reflex was discovered (Tracey, 2002), quantification of the HRV components, which are not directly related to respiration, is important for the analysis of long-range and scaling properties of the cardiac dynamics (Ivanov et al., 1999; Schmitt and Ivanov, 2007). Examples of application of RSA analysis include clinical psychology (Wielgus et al., 2016), treatment of substance use disorder (Price and Crowell, 2016), prediction of the course of depression (Panaite et al., 2016), quantification of cardiac vagal tone and its relation to evolutionary and behavioral functions (Grossman and Taylor, 2007), quantification of vagal activity during stress in infants (Ritz et al., 2012), and even in cancer patients (Moser et al., 2006), to name just a few.

A variety of data analysis techniques quantifying RSA have been proposed in the literature, for a discussion of commonly used metrics and their advantages and drawbacks see, e.g. (Lewis et al., 2012). The techniques quantifying RSA can be divided into two categories, the time domain and the frequency domain. In time domain, continuous wavelet transform (WTC) is used for its advantage to analyze transient and non-linear signals. This method demonstrates the dynamic behavior of respiration sinus arrhythmia through the analysis of the WTC between heart rate and respiration signals (Jan et al., 2019). Phase analysis technique could help to disentangling respiratory sinus in heart rate variability records, but the final HRV index obtained by this technique is complex to calculate in time domain, and its physiological significance is not clear (Topcu et al., 2018). There are works which use respiratory and RR sequences to calculate G-causality and system gain as the measure of RSA. Much further work is needed to make these produced measures useful in clinical research and applications (Fonseca et al., 2013). In the frequency domain, HF of HRV indicators quantifies RSA on specific frequency bands, while Cardiopulmonary Coupling (CPC) measures the correlation between RR interval and respiratory sequence in the frequency domain. Both are empirical, without solid theoretical foundation and systematic design, therefore serious clinical applications are not seen so far (Thomas et al., 2005).

In the rest of this article, our contributions in developing quantitative measures for RSA are described as follows:

In section Cardiopulmonary Resonance Model (CRM), we model RSA as modulation of heart rate by respiration in an interactive cardiopulmonary system with the most effective system state of resonance. Mathematically, it is described by bivariate autoregressive model of respiration series and RR intervals, and quantitatively it is assessed by Granger causality function. The whole model is referred to as Cardiopulmonary Resonance Model (CRM).

In section Cardiopulmonary Resonance Indices (CRI), based on the cardiopulmonary resonance concept, and Granger causality function which is referred to as cardiopulmonary resonance function (CRF) after, a set of quantitative measures for RSA is proposed, and named as Cardiopulmonary Resonance Indices (CRI).

In section Applications Scenarios, to show the effectiveness of CRM and CRI, two application scenarios, paced breathing and sleep stage discrimination, are studied. It has been shown that CRF and CRI provide ideal visual interpretation and numerical measures for cardiopulmonary interactions toward resonance state in paced breathing scenario as the paced breathing rate coming down to 0.1 Hz. The same is true as the sleep stage moves to deep sleep.

## Cardiopulmonary Resonance Model (CRM)

We are committed to building a cardiopulmonary resonance model for the purpose of quantitative assessment of RSA with hypothesis that cardiopulmonary interaction is important in circulation system to ensure efficient delivery of oxygen and nutrient, and that the efficiency is optimized at the state of cardiopulmonary resonance. Mathematically, we present a bivariate autoregressive model of respiration series and RR intervals, calculate respiratory and non-respiratory related component on RR intervals in the frequency domain using Granger-causality.

### Bivariate Autoregressive Model of Respiration Series and RR Intervals

The cardiopulmonary interaction can be interpreted as functional connectivity analysis such as synchrony (Engel and Singer, 2001) and phase coherence (Nunez et al., 2001) and so on. Our model takes direct central respiratory modulation of the parasympathetic cardiac signal as the main mechanism for RSA. A powerful technique for extracting directed functional connectivity from data is Granger causality (G-causality) (Granger, 1969). According to G-causality, *X*_2_causes *X*_1_ if the inclusion of past observations of *X*_2_ reduces the prediction error of *X*_1_ in a linear regression model of *X*_1_ and *X*_2_, as compared to a model which includes only previous observations of *X*_1_.

The change process of RR can be regarded as a Markov process, ignoring other factors affecting heart rate in short term, we described the RR intervals(*X*_1_(*t*)) and respiration signal(*X*_2_(*t*)) (both of length T) by a bivariate auto-regressive model:

$$\begin{array}{r} \begin{array}{c} X_{1}\left(t\right)=\overset{p}{\underset{j=1}{\sum }}A_{11,j}X_{1}\left(t-j\right)+\overset{p}{\underset{j=1}{\sum }}A_{12,j}X_{2}\left(t-j\right)+\xi_{1}\left(t\right)\\ X_{2}\left(t\right)=\overset{p}{\underset{j=1}{\sum }}A_{21,j}X_{1}\left(t-j\right)+\overset{p}{\underset{j=1}{\sum }}A_{22,j}X_{2}\left(t-j\right)+\xi_{2}\left(t\right) \end{array} \end{array}$$

where *p* is the maximum number of lagged observations included in the model (the model order, *p* < T). *A* contains the coefficients of the model, and ξ_1_, ξ_2_ are the residuals for each time series.

In order to ensure RR intervals in the normal range and without a mutation, we use interpolation as a substitute for points that do not meet the following conditions:

$$\begin{array}{r} \;|\;RRI_{i}-\bar{RRI}|<1.5*Std\left(RRI\right)\\ 0.7*RRI_{i-1}<RRI_{i}<1.3*RRI_{i-1} \end{array}$$

where *RRI* is the RR intervals, *RRI*_*i*−1_and *RRI*_*i*_ are adjacent intervals.

For each record around 120 s, under the assumption of stationary property of signals, and for efficiency of computation, we normalize the RR intervals and respiration series to zero-mean and unit variance. The magnitude of RSA can be measured by the log ratio of the prediction error variances for the restricted (R) and unrestricted (U) models:

$$\begin{array}{r} G_{2\to 1}=\ln\;\frac{\mathrm{var}\left(\xi_{1R\left(12\right)}\right)}{\mathrm{var}\left(\xi_{1U}\right)} \end{array}$$

where ξ_1*R*(12)_ is derived from the model omitting the *A*_12, *j*_(for all j) coefficients in the first equation and ξ_1*U*_is derived from the full model.

The estimation of the model of each record requires as a parameter the number of time-lags (*p*) to include, i.e., the model order. A principle means to specify the model order is to minimize a criterion that balances the variance accounted for by the model, against the number of coefficients to be estimated. We chose the Akaike information criterion (Akaike, 1974) for *n* variables in which the ∑ denotes the noise covariance matrix:

$$\begin{array}{r} AIC\left(p\right)=\ln\;\left(\det\left(\sum \right)\right)+\frac{2pn^{2}}{T} \end{array}$$

### Spectral G-causality of Respiration Series and RR Intervals

For the dynamics of the cardiopulmonary system are easier to understand and interpret in the frequency domain, we calculate the Spectral G-causality of respiration series and RR intervals.

The Fourier transform of the bivariate auto-regressive model in time domain gives:

$$\begin{array}{r} \left(\begin{array}{cc} A_{11}\left(f\right) & A_{12}\left(f\right)\\ A_{21}\left(f\right) & A_{22}\left(f\right) \end{array}\right)\left(\begin{array}{c} X_{1}\left(f\right)\\ X_{2}\left(f\right) \end{array}\right)=\left(\begin{array}{c} E_{1}\left(f\right)\\ E_{2}\left(f\right) \end{array}\right) \end{array}$$

in which the components of *A* are

$$\begin{array}{r} A_{lm}\left(f\right)=\delta_{lm}-\overset{p}{\underset{j=1}{\sum }}A_{lm}\left(j\right)e^{\left(-i2\pi fj\right)},\\ \delta_{lm}=0\left(l=m\right),\delta_{lm}=1\left(l\neq m\right) \end{array}$$

*E* is the Fourier transform of the residual matrix.

For the sake of calculation, we rewrite it as

$$\begin{array}{r} \left(\begin{array}{c} X_{1}\left(f\right)\\ X_{2}\left(f\right) \end{array}\right)=\left(\begin{array}{cc} H_{11}\left(f\right) & H_{12}\left(f\right)\\ H_{21}\left(f\right) & H_{22}\left(f\right) \end{array}\right)\left(\begin{array}{c} E_{1}\left(f\right)\\ E_{2}\left(f\right) \end{array}\right) \end{array}$$

where *H* is the transfer matrix. The spectral matrix *S* can now be derived as

$$\begin{array}{r} S\left(f\right)=\langle X\left(f\right)X^{*}\left(f\right)\rangle =\langle H\left(f\right)\sum H^{*}\left(f\right)\rangle \end{array}$$

in which the ∑ denotes the noise covariance matrix.

*A* split of U into sub-processes *X* and *Y* includes a decomposition

$$\begin{array}{r} S\left(f\right)=\left(\begin{array}{cc} S_{xx}\left(f\right) & S_{xy}\left(f\right)\\ S_{yx}\left(f\right) & S_{yy}\left(f\right) \end{array}\right) \end{array}$$

of the cross-power spectral density and a similar decomposition for the transfer function *H(f)*.

Then *S*_*xx*_(*f*) is the spectral density of *X*, which is given by

Sxx(f)=Hxx(f)∑xxHxx*(f)+2Re{Hxx(f)              ∑xyHxy*(f)}+Hxy(f)∑yyHxy*(f)

Thus, we can get the Spectral G-causality of respiration series and RR intervals:

$$\begin{array}{r} G_{Y\to X}\left(f\right)=\ln\;\left(\frac{\left|S_{xx}\left(f\right)\right|}{\left|S_{xx}\left(f\right)-H_{xy}\left(f\right)\sum_{y|x}H_{xy}{\left(f\right)}^{*}\right|}\right) \end{array}$$

$$\begin{array}{r} \sum_{y|x}\equiv \sum_{yy}-\sum_{yx}\sum_{xx}^{-1}\sum_{xy} \end{array}$$

where ∑denotes the residual covariance matrix.

For the non-respiratory components, we get:

$$\begin{array}{r} G_{N-RESP}\left(f\right)=1\text{-}G_{Y\to X}\left(f\right) \end{array}$$

Now we have both the measurement of respiratory and the non-respiratory components effects on RR intervals in the frequency domain, R-HRV and NR-HRV, respectively. Here we focus on the spectral G-causality of respiration series and RR intervals, *G*_*Y*→*X*_*(f)*. For convenience, we simply write it as *G(f)*, and rename it as Cardiopulmonary Resonance Function (CRF) in the rest of this article.

## Cardiopulmonary Resonance Indices (CRI)

With cardiopulmonary resonance function (CRF), we are now able to establish a quantitative measurement for RSA, referred to as Cardiopulmonary Resonance Indices (CRI), with the hope that it will be able to play a role in quantifying cardiopulmonary system efficiency, and as a biomarker for cardiac vagal tone and well-being, on the basis of CRF and key concept of cardiopulmonary resonance.

**Figure 1:**
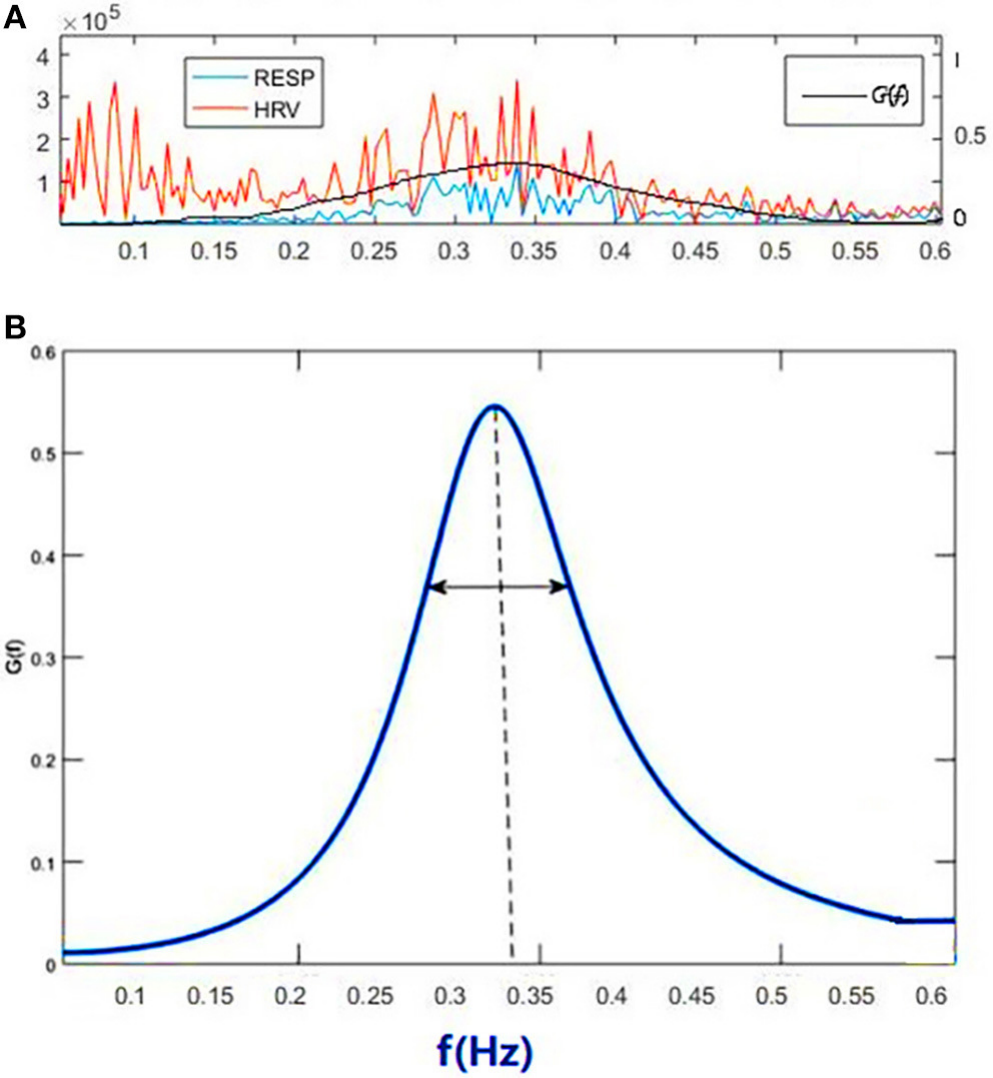
Illustration of Cardiopulmonary Resonance Function (CRF) and Cardiopulmonary Resonance Indices (CRI). **(A)** the power spectral curves of RR interval series and respiration series, as well as the corresponding cardiopulmonary resonance function, *G(f)*. **(B)** the schematic diagram of CRA and CRW. CRA is taken from the maximum point of *G(f)* (CRF); CRW, the bandwidth of CRF as indicated by the bi-directional arrow line.

Figure 1A shows the power spectral curves of RR interval series and respiration series, as well as the corresponding cardiopulmonary resonance function, *G(f)*. *G(f)* represent the strength of RSA, the modulation of respiration to heart rate. *G(f)* is a monotonic function of frequency *f* with single peak around the main respiration frequency, can be considered as the spectral energy distribution function of cardiopulmonary resonance system. The cardiopulmonary resonance indices (CRI) consists of the following numerical measure:

A) Cardiopulmonary Resonance Amplitude (CRA) is defined as the maximum of Cardiopulmonary resonance function *G(f)*:

$$\begin{array}{r} CRA\equiv M\text{ax}CRF \end{array}$$

Refer to Figure 1B, in consideration of the main frequency bands of heart rate variability and respiration rate, *G(f)* is plotted in the frequency range of 0.0033–0.5 Hz. Denote the frequency where the maxima of *G(f)* appears as cardiopulmonary resonance frequency *f*_*A*_. CRF is around main respiration rate. In free breathing, respiration rate is around 0.20–0.30 Hz, in the range of HRV high frequency. That is the point of consistence between HRV_HF measure and RSA strength in representing the regal level. As we will see in the next section paced breathing experiments, as paced breathing frequency down to 0.1 Hz, both RSA energy and HRV energy shall move and focus around 0.1 Hz as well. In this case, the hypothesis of HRV_HF representing regal activity may not hold.

B) Cardiopulmonary Resonance bandWidth (CRW). As shown in Figure 1B, CRW is defined as the CRF bandwidth, the degree of RSA energy concentration. CRA and CRW are related. While CRW is narrow, CRA is big.

C) Cardiopulmonary Resonance Quality factor (CRQ). CRQ is defined to measure the merit of the cardiopulmonary resonance system by adopting the quality factor measure for inductor, capacitor, and resistor LCR oscillator where interaction between lung and heart resemble the energy flow between inductor and capacitor, while non-respiration factors are equivalent to resistor, damping the resonance. Mathematically, CRQ is defined as

$$\begin{array}{r} CRQ=\frac{f_{A}}{CRW} \end{array}$$

Considering the physiological functions, RSA serves to minimize the energy expenditure of the heart while keeping arterial CO_2_ levels at physiological tensions. CRQ measures the energy conversion of the system. The lower the dissipation energy, the higher the quality factor and metabolic efficiency. High CRQ indicates high efficiency of cardiopulmonary metabolic system and relatively healthy physiological and psychological state.

## Applications Scenarios

In this section, two application scenarios are presented to demonstrate the descriptive power of CRF and CRI, as well as the application potentials.

### Paced Breathing

#### Experiment Design

HRV biofeedback has been used for the treatment of depression and other autonomic related problems. HRV biofeedback uses HRV measures, mainly time domain and frequency domain, as feedback cues to guide the subject performing slow paced breathing in order to reach resonance state. The objectives of paced breathing in HRV biofeedback is to gain level of parasympathetic nerves activity and improve the autonomic balance. As such, the measures of current status of the subject play most important role in biofeedback process. So far in the HRV biofeedback HRV measures are used, while HRV measures have problems in representing autonomic regulation status (Vaschillo et al., 2006).

The essential physiological phenomenon of the slow and deep paced breathing in HRV biofeedback is respiration sinus arrhythmia (RSA). The level of RSA should be the natural measure as biofeedback cues. As the quantitative measure of RSA, CRF and CRI provide the best visual cue and numerical cues for biofeedback.

During the paced breathing, Cardiopulmonary Resonance Amplitude (CRA) could help us find the optimal respiratory rate for individuals which is usually around 0.1 Hz. The process of training is the process of making CRA keep approaching 1. As we go from the resting state to paced breathing rate coming down to 0.1 Hz, with the frequency decreases, CRA gets bigger and the bandWidth CRW gets smaller. The frequency of obtaining the maximum value of CRA is the personalized resonance frequency of the subject and also the frequency of biofeedback. As an indicator of cardiopulmonary system metabolism, cardiopulmonary resonance quality factor (CRQ) indicates efficiency of cardiopulmonary metabolic system and relatively healthy physiological and psychological state.

This study was carried out in accordance with the recommendations of guidelines of ethical review of clinical research ethics committee of China-Japanese Friendship Hospital. The number is 2019-GZR-138. The protocol was approved by the clinical research ethics committee of Beijing China-Japanese Friendship Hospital. All participants signed informed consent forms. We collected data from 30 healthy adults in ages of 20–30. The baseline demographic characteristics of 30 participants are shown in Table 1. The subjects' age, height, weight, and mean systolic and diastolic blood pressure were counted and presented as mean ± standard deviation. The paired *t*-test showed there were no significant differences in age, systolic and diastolic blood pressure between the male and female groups.

**Table 1:** Baseline Demographic Characteristics of 30 Participants and *p*-value between 15 men and 15 women.

**Characteristics**	**Men(15)**	**Women(15)**	***p***
Age (y)	24.40 ± 2.830	23.50 ± 2.134	0.631
Height (cm)	175.53 ± 4.872	163.31 ± 4.457	0.035
Weight (kg)	68.33 ± 8.205	57.81 ± 6.304	0.025
SBP (mm Hg)	110.50 ± 9.375	109.90 ± 6.845	0.302
DBP (mm Hg)	68.30 ± 6.521	64.40 ± 8.347	0.413

*Data presented as mean ± standard deviation*.

**Figure 2:**
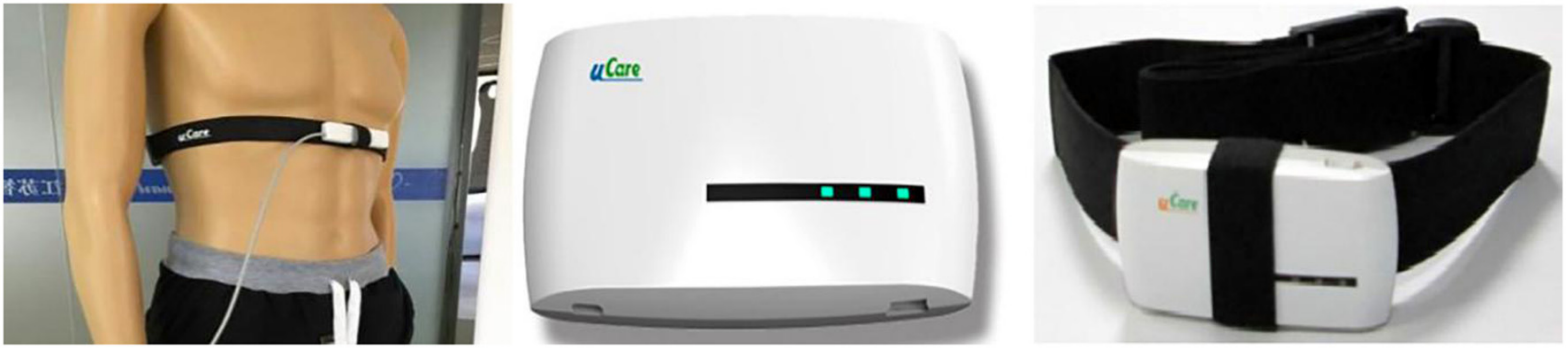
The wearable hardware device used to collect ECG and respiration signals.

The data is collected using one intelligent hardware, worn on the wrist (Figure 2). We collected one-lead ECG and respiratory signals of everyone from resting to biofeedback status. The whole process is recorded. During the process of paced breathing rate down to about 0.1 Hz, we use our method to find the individual resonant frequency for every trainee: Starting from the resting state of the subjects, the breathing rate was gradually reduced at 0.01 Hz intervals guided by voice and image on the computer. Each breathing rate was maintained for at least 1 min. It can be seen that Cardiopulmonary Resonance Amplitude (CRA) gradually increases as the respiratory rate decreases and reaches its maximum value around 0.1 Hz. The frequency of obtaining the maximum value of CRA is the personalized resonance frequency of the subject and also the frequency of biofeedback.

#### Statistical Analysis

To demonstrate the advantage of CRI in paced breathing compared to HRV, we calculated the Cardiopulmonary Resonance Indices (CRI) and HRV in different statues. In order to represent CRF and CRA visually, we draw the CRF curves in the frequency domain with HRV in four status of paced breathing from resting status to biofeedback status. The repeated one-way ANOVA, followed by Dunnett's *post hoc* test was used to represent the significant difference from resting state to biofeedback state of CRI in the breathing training.

#### CRF and CRI in Paced Breathing

**Figure 3:**
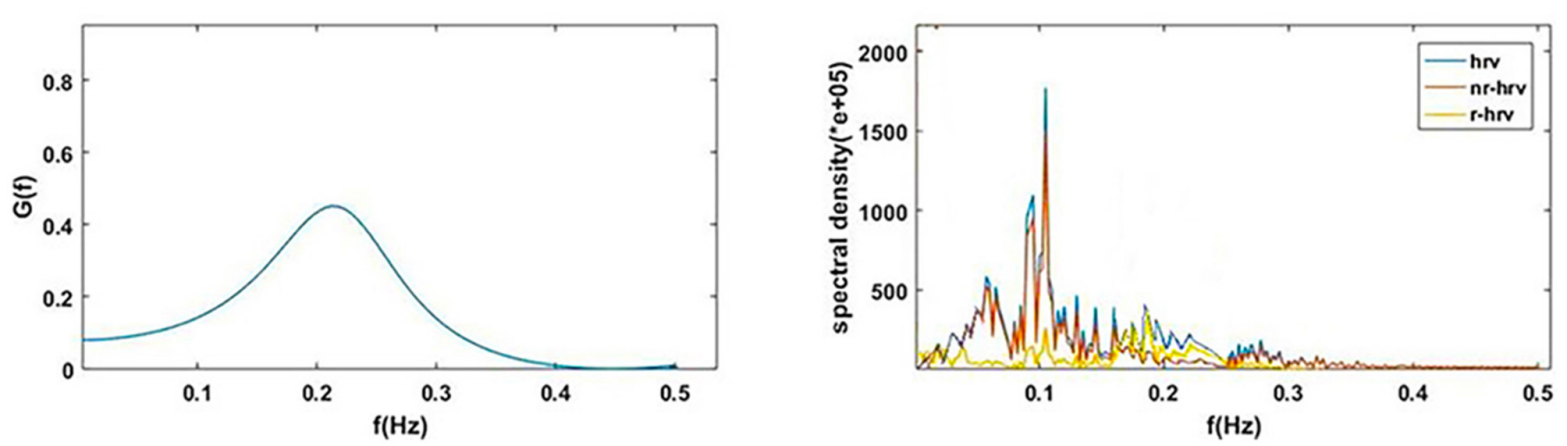
*G(f)* (CRF) and corresponding HRV, R-HRV, and NR-HRV in frequency domain.

CRF measures the effect of respiration on current heart rate changes in the frequency domain. CRF and corresponding HRV, R-HRV, and NR-HRV in the frequency domain are shown in Figure 3.

Respiratory effects in different physiological states have different effects on heart rate. These effects can be directly seen from the power spectrum calculated by spectral G-causality, which is closely related to the current breathing rate of the subjects. CRF expresses the cardiopulmonary interaction at the current time in the frequency domain.

**Figure 4:**
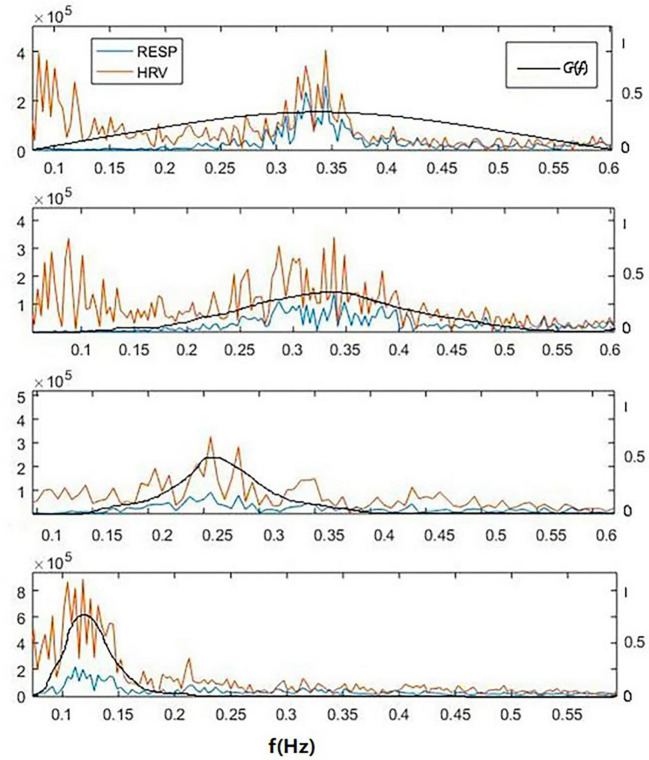
A typical power spectral density curves of respiratory, HRV and corresponding cardiopulmonary resonance function CRF in the frequency domain of a subject for 4 cases of breathing at resting state. From the top: free breathing, paced breathing at frequency of 0.33, 0.26, and 0.12 Hz. It can be seen that paced breathing increases the strength of RSA, and that as the frequency of paced breathing coming down toward, the cardiopulmonary resonance phenomenon becomes stronger, which is very well-captured by the cardiopulmonary resonance function. It can also seen that as the paced breathing frequency approaches 0.1 Hz, around which there is an optimal resonance state for the subject, where HRV_HF is small. That is to say that CRI do represent level of parasympathetic nervus activity at various cases, while HRV do not.

To demonstrate the advantage of CRI in paced breathing compared to HRV, the CRF, HRV, and respiratory power spectral density of one subject of the 30 participants in the experiment from resting to biofeedback status are illustrated in Figure 4. The blue lines show respiratory power spectral density, orange lines show HRV and black lines show CRF. From the top: free breathing, paced breathing at frequency of 0.33, 0.26, and 0.12 Hz. It can be seen that paced breathing increases the strength of RSA, and that as the frequency of paced breathing coming down toward, the cardiopulmonary resonance phenomenon becomes stronger, which is very well-captured by the cardiopulmonary resonance function. We can also see that as the paced breathing frequency approaches 0.1 Hz, around which there is an optimal resonance state for the subject, where HRV HF is small. That is to say that CRI do represent level of parasympathetic nervus activity at various cases, while HRV do not.

The higher degree of cardiopulmonary coupling during paced breathing, the respiration accounts for a higher proportion of HRV. In the resting state, HRV produced by breathing is weak, then NR-HRV can reflect the influence of other physiological activities on heart rate through autonomic nervous activity.

As can be seen from the figure, with the change of respiratory rate, the distribution of CRF and HRV both shift in the corresponding frequency bands. The distribution of the frequency band of HRV is closely related to the change of respiratory frequency, so the degree of biofeedback can be observed from respiration frequency shift (Vaschillo et al., 2006). However, there is no quantitative measure between the peak value of HRV and the respiratory frequency within HRV biofeedback to describe the intensity and depth of cardiopulmonary interaction. Meanwhile, HRV cannot be used as a measure of RSA due to its low repeatability and large individual differences.

On contrary, CRF show a clear trend in paced breathing. Generally, we calculated CRI in four different states from resting to biofeedback status (from 1 to 4) in Table 2. The repeated one-way ANOVA was used to test the differences of CRI in the four states (*p* < 0.05). In Table 2, the *p*-values of CRA, CRW and *f*_*A*_are smaller than 0.05, and the *p*-value of CRQ is bigger than 0.05. The results showed that CRA, CRW and *f*_*A*_ have significant differences in the four status. CRQ is defined to measure the merit of the cardiopulmonary resonance system, and did not change significantly at different respiratory rates. In order to confirm the significance and stability of the differences between CRA and CRW in the four states further, we performed Dunnett's *post hoc* test shown in Table 3. As we can see, CRA increases and CRB decreases during the training. In the most of the pairwise comparisons of state 1, 2, 3, and 4, CRA and CRW show significant differences. These two indicators together represent the intensity of a person's cardiopulmonary interaction and reflect the activity and regulatory capacity of the human vagus nerve with repeatability and stability.

**Table 2:** Cardiopulmonary Resonance Indices for the 4 cases of breathing: free breathing and 3 paced breathing at frequency of 0.33, 0.26, and 0.12Hz.

	**1**	**2**	**3**	**4**	**p**
CRA	0.640 ± 0.004	0.710 ± 0.003	0.810 ± 0.005	0.991 ± 0.004	0.003
CRW	0.250 ± 0.030	0.170 ± 0.021	0.130 ± 0.020	0.075 ± 0.021	0.004
*f_*A*_*	0.360 ± 0.030	0.301 ± 0.030	0.201 ± 0.021	0.110 ± 0.020	0.002
CRQ	1.440 ± 0.375	1.760 ± 0.313	1.541 ± 0.240	1.470 ± 0.304	0.146

*The p-value represents the result of the a repeated measures one-way ANOVA. P values show that there is a significant difference between the groups in CRA, CRW and f_A_ (P < 0.05)*.

**Table 3:** Dunnett's *post-hoc* test of CRA and CRW for the 4 cases of breathing: free breathing (1), and 3 paced breathing at frequency of 0.33 Hz (2), 0.26 Hz (3), and 0.12 Hz (4).

**Comparative**	**CRA**		**CRW**	
**group**				
	**Difference of**	**LSR (*p* = 0.05)**	**Difference of**	**LSR(*p* = 0.05)**
	**the mean**		**the mean**	
4 and 1	0.351	0.097	0.175	0.047
4 and 2	0.281	0.096	0.095	0.045
4 and 3	0.181	0.096	0.055	0.045
3 and 1	0.170	0.095	0.120	0.042
3 and 2	0.100	0.095	0.040	0.040
2 and 1	0.070	0.095	0.080	0.040

*If the value of Difference of the mean > LSR, there is a significant difference between the groups being compared (p < 0.05)*.

We can see that CRF and CRI could provide ideal visual interpretation and numerical measures for cardiopulmonary interactions toward resonance state in paced breathing scenario. CRF can be used to analyze the human body in different physiological states, get cardiopulmonary coupling value accurately, and analyze the regulation process of human sympathetic and parasympathetic nerves.

### Sleep Stage Discrimination

#### Experiment Design

Cardiopulmonary Coupling index (CPC) was proposed by Thomas et al. (2005) in 2005, which measures the spectral correlation between heart rate sequence and respiratory signal. Therefore, CPC can be a candidate providing measures for RSA. CPC is defined as the product of the average cross-spectral power divided by the average power of each signal as below.

$$\begin{array}{r} CPC\left({\text{f}}_{n}\right)={\langle \Gamma_{n}\left(R,E\right)\rangle }^{2}\Lambda_{n} \end{array}$$

$$\begin{array}{r} \Lambda_{n}=\frac{{\langle \Gamma_{n}\left(R,E\right)\rangle }^{2}}{\langle {\hat{R}}_{n}^{2}\rangle \langle {Ê}_{n}^{2}\rangle } \end{array}$$

in which Γ_*n*_(*R, E*) denotes the cross spectrum of RR intervals and respiratory signals. CPC reflects the degree of sleep and respiratory rhythm disorder through the high frequency, low frequency and very low-frequency parts with a different energy. It overcomes the shortcomings and defects of the HRV method used in the analysis alone. At present, this method has been widely used in the field of evaluating sleep quality and judging sleep and breathing disorders (Yang et al., 2011).

In sleep stage discrimination, we used data from the MIT-BIH database (Ichimaru and Moody, 1999) which has sleep stage labels from polysomnography (PSG). CRI in different sleep stage was calculated and a comparative study was conducted by using Cardiopulmonary Coupling index (CPC) in sleep stage classification. Except for the heart rate, HRV, and respiratory rate, CRA, CRW, and *f*_*A*_ extracted from CRF, meanwhile LF, HF, and LF/HF extracted from CPC are, respectively, used in the classification task to test sleep stage of the subject.

**Figure 5:**
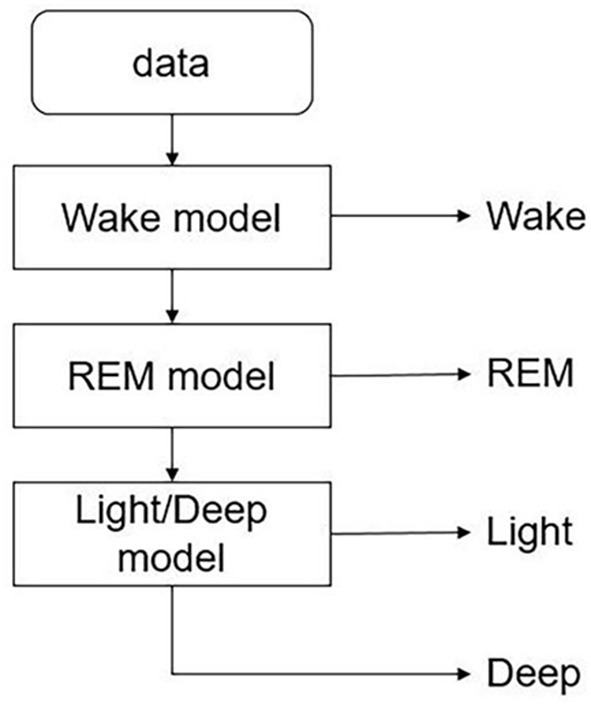
DTB-SVM model for classifying the sleep stages. First, a classifier is used to separate the awake phase and the sleep phase, and then within the sleep phase, the REM phase and the NREM phase are separated, and finally the light sleep and deep sleep are separated by the last classifier.

In the experiment, the classifier needs to identify four different sleep stages, including awake, REM, light sleep, and deep sleep. SVM is not able to solve multi-category classification problems directly, but the combination of SVM and decision tree (called DTB-SVM) can be used to solve multi-class classification problems. Based on the structural characteristics of the sleep cycle and the physiological features used in sleep classification, we used three SVM models to classify the sleep stages. As shown in Figure 5, firstly, a classifier is used to separate the awake phase and the sleep phase, and then within the sleep phase, the REM phase and the NREM phase are separated, and finally the light sleep and deep sleep are separated by the last classifier. RBF kernel was used in the model. In order to prevent over-fitting, we selected the optimal parameters in the way of K-fold cross-validation and grid search. For the features of CPC and CRI, classifiers of same structure were used to classify the sleep stages.

#### Statistical Analysis

The confusion matrix was used to explain the accuracy of sleep classification results and to compare the performance of CRI and CPC in classification tasks. Each row of the matrix represents the prediction category, and the total number of each row represents the number of data predicted for that category. Each column represents the true category to which the data belongs, and the total number of data in each column represents the number of data instances in that category.

The effectiveness of the features was measured by the ratio of intra-class divergence and inter-class divergence. The ratio between intra-class divergence and inter-class divergence is defined and calculated as follows (Zhou et al., 2010):

Let (*X, y*)ϵ(R^n^ × y) be a sample, where R^n^ is an n-dimensional feature space and *y* = {1,2,.,s} is the label set. *L*_*i*_ is the number of samples in the *i*th class, and *l* is the total number of samples. Let *X*_*ij*_ denote the *j*th sample in the *i*th class, *m*_*i*_ the sample mean of the *i*th class, and *m* the sample in the *i*th class, *m*_*i*_ the sample mean of the *i*th class, and m the sample mean of all class. The within-class scatter matrix *S*_*W*_, between-class scatter matrix *S*_*B*_ are defined as

$$S_{w}=\sum_{i=1}^{s}\sum_{j=1}^{l_{i}}(X_{ij}-m_{i})(X_{ij}-m_{i}{)}^{T}$$

$$S_{B}=\sum_{i=1}^{s}l_{i}\left(m_{i}-m\right){\left(m_{i}-m\right)}^{T}$$

Large class separability means small within-class scattering and large between-class scattering. A combination of two of them can be used as a measure, |*S*_*W*_ |/|*S*_*B*_|, where |·| denote the determinant of a matrix. The smaller the ratio, the better the effect of the feature on classification.

In order to demonstrate the good performance of CRI in sleep classification task, we compared the confusion matrix of CRI and CPC classification results. Then, in order to express the role of features further, we conducted the divergence analysis on the features of CRI and CPC. The results showed that CRI was more effective than CPC in sleep classification task, especially in the deep sleep recognition.

#### CRI in Different Sleep Stages Compared to CPC

**Figure 6:**
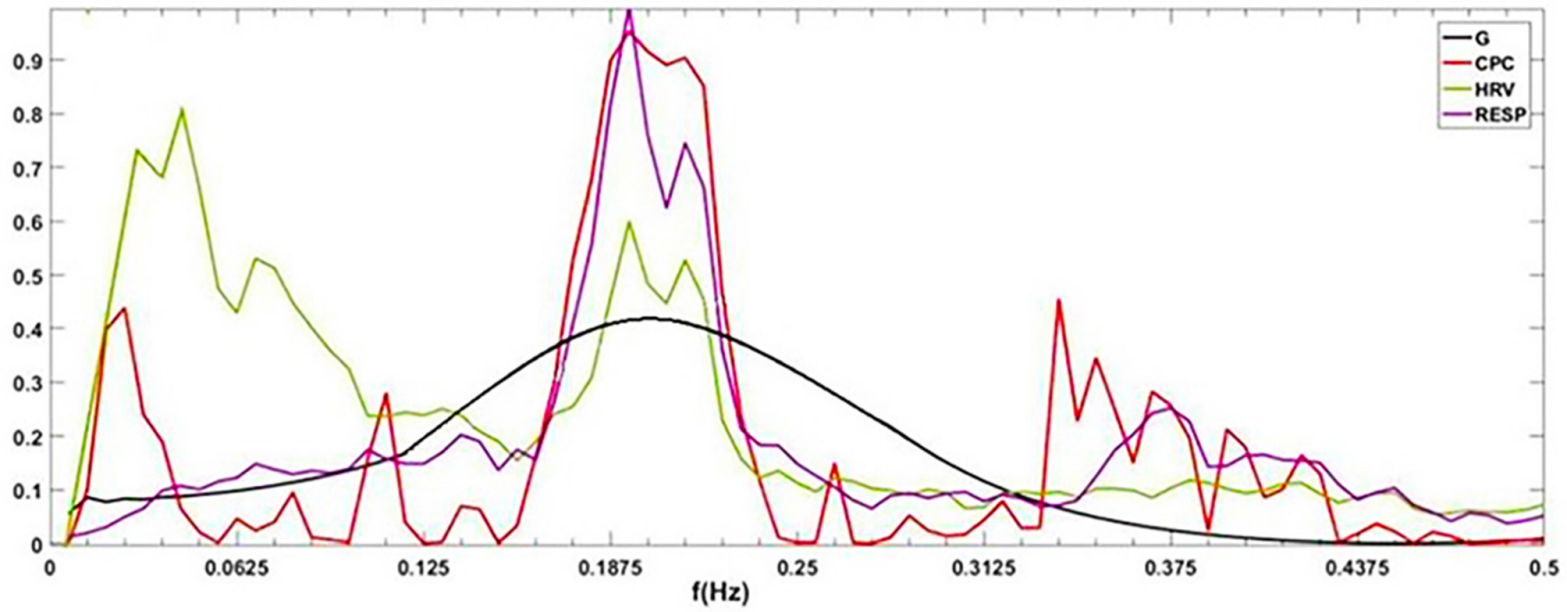
*G(f)* (CRF), CPC, power spectral density of HRV and respiration. By definition, cardiopulmonary resonance function CRF, reflects the strength of RSA, with the peak near the mean of the respiration rate, while CPC is the correlation between HRV and respiration with respect to their power spectrum, having multiple peaks. As the name indicated, CRF well capture the resonance nature of cardiopulmonary system.

To visually compare the difference between CRI and CPC, Figure 6 shows the CPC, HRV, and CRF of one subject. It contains HRV (0.14 Hz as the demarcation line between high frequency and low frequency), respiration power spectral density, CPC index and *G(f)*, which are all discussed in the frequency domain.

CPC represents the correlation of RR intervals and respiratory signal. It shows that CPC has multiple peaks in the full frequency band. In low-frequency band, for HRV analysis, people usually think sympathetic nerve and parasympathetic nerve interact together, and CPC also shows a spike which indicates a high correlation between respiration and RR intervals, such as blood pressure, etc. It is difficult to find an exact indicator representing the cardiopulmonary coupling state from CPC. Physiologically, RSA, the strength of respiration modulation of heart rate should appear as CRF, cannot be multiple peaks as CPC.

The indices of one subject in four different sleep stage of one night are shown in Table 4. It shows that our indices could express the cardiopulmonary interaction phenomenon and the degree of cardiopulmonary coupling resonance in different sleep stages. Table 5 shows the performance of CRI indicators and CPC indicators on the whole data set in the classification task. CRW, CRA, *f*_*A*_are smaller than LF_CPC and HF_CPC. The results show that the CRI features including CRW, CRA, *f*_*A*_ performed better than CPC features including LF_CPC and HF_CPC in the classification task.

**Table 4:** Cardiopulmonary Resonance Indices of one subject in different sleep stages of a whole night.

	**Wake**	**REM**	**Light**	**Deep**
CRA	0.648 ± 0.004	0.674 ± 0.004	0.734 ± 0.003	0.993 ± 0.004
CRW	0.280 ± 0.011	0.231 ± 0.011	0.163 ± 0.010	0.053 ± 0.013
*f_*A*_*	0.300 ± 0.020	0.290 ± 0.017	0.25 ± 0.016	0.230 ± 0.010
CRQ	1.071 ± 0.230	1.255 ± 0.227	1.534 ± 0.161	4.340 ± 0.102

*Data presented as mean ± standard deviation*.

**Table 5:** The divergence analysis of the features of CPC and CRI in the sleep classification task.

	**LF_CPC**	**HF_CPC**	**CRW**	**CRA**	***f_***A***_*_**_**_CRI**
|*S_*B*_* |/|*S_*W*_*|	7.290	5.365	0.302	1.930	2.311

*LF_CPC and HF_CPC are the LF and HF features of CPC, CRW, CRA, and fA_CRI are the features of CRI*.

The classification results of CRI and CPC were statistically analyzed, respectively, in Table 6. It shows that CRI shows superior in distinguishing deep sleep stage than CPC. The overall accuracy of the classification went up 1.28%. Particularly shown in Table 6, great progress has been made in distinguishing between deep sleep and light sleep, and the recognition rate of deep sleep has been increased by 11.35%. It shows that CRF performances better than CPC, especially in the distinction between light sleep (NREM_1 and NREM _2) and deep sleep (NREM_3 and NREM _4).

**Table 6:** Confusion matrix of sleep stage classification using CPC and CRI.

**Actual predicted**	**Wake**		**REM**		**Light**		**Deep**	
	**CPC**	**CRI**	**CPC**	**CRI**	**CPC**	**CRI**	**CPC**	**CRI**
Wake	1557	1561	44	44	305	307	50	25
REM	27	30	356	355	97	100	8	12
Light	219	220	88	89	3994	4034	115	87
Deep	13	5	5	5	77	32	259	308
Total	1816	1816	493	493	4473	4473	432	432
Accuracy	85.74%	85.96%	72.21%	72.01%	89.29%	90.19%	59.95%	71.30%

*Each value in the table represents the number of samples. At each position in the obfuscation matrix, the values on the left represent the results of CPC, and the values on the right represent the results of CRI. CRI shows superiority in identifying deep sleep, more than 11% higher than CPC*.

**Figure 7:**
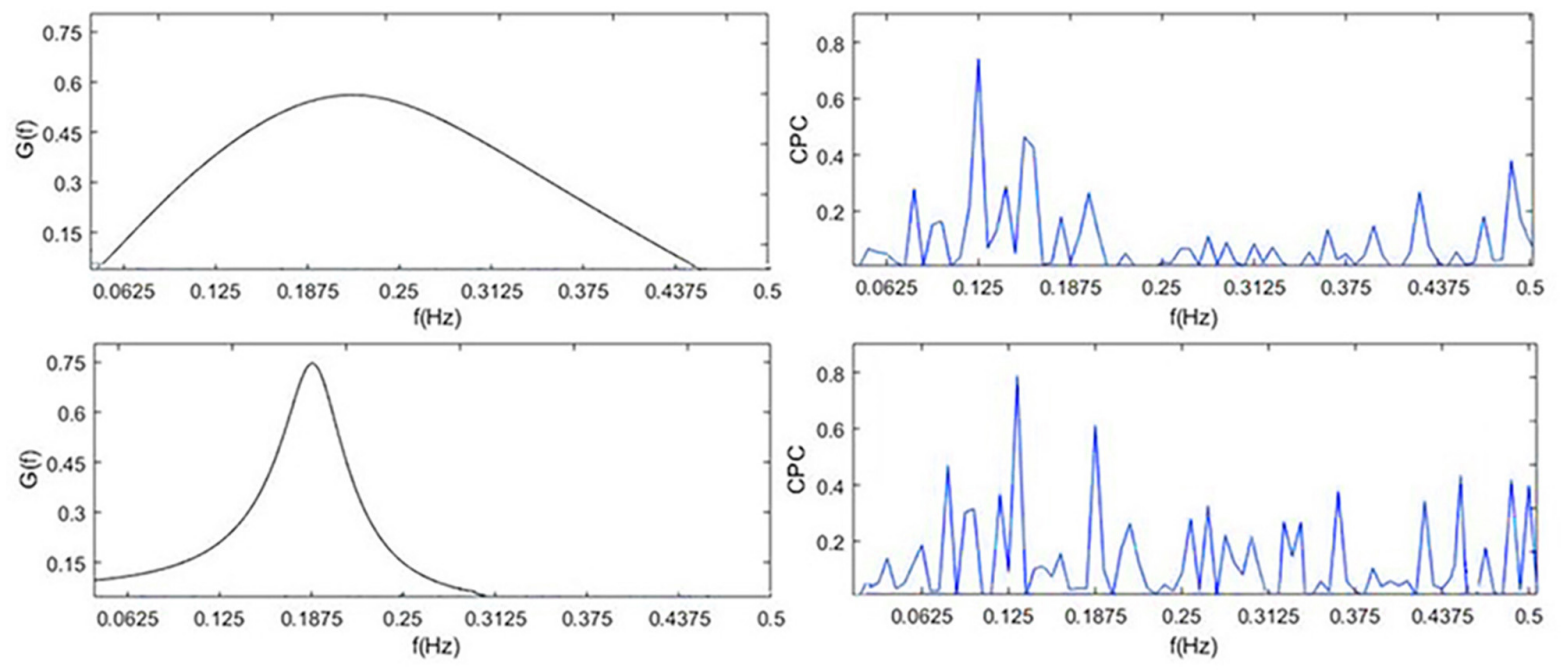
*G(f)* (CRF) and CPC in light sleep (top) and deep sleep (bottom).

The performance of CPC and CRF in deep sleep and light sleep is shown in Figure 7. In the deep sleep stage, CRF shows more concentrated and indicators we proposed are good indications of this phenomenon. It provides meaningful features for the distinguishing of the two. To illustrate the role of CRI in deep sleep recognition, divergence analysis of the features of CPC and CRI in the deep sleep and light sleep was performed in Table 7. It shows the performance of CRI indicators and CPC indicators for distinguishing the deep sleep stage. CRW, CRA, *f*_*A*_are much smaller than LF_CPC and HF_CPC. It shows that CRI features CRW, CRA, *f*_*A*_ performed much better than CPC features LF_CPC and HF_CPC. In the deep sleep, the cardiopulmonary system has the highest metabolic efficiency and the smallest dissipated energy, and the body and mind of the human body can fully rest and recover. This suggests that CRI is a good indicator for different sleep status especially the deep sleep of human body.

**Table 7:** The divergence analysis of the features of CPC and CRI in the deep sleep and light sleep.

	**LF_CPC**	**HF_CPC**	**CRW**	**CRA**	***f_***A***_*_**_**_CRI**
|*S_*B*_* |/|*S_*W*_*|	8.312	5.432	0.530	1.106	2.867

*LF_CPC and HF_CPC are the LF and HF features of CPC, CRW, CRA, and f_A__CRI are the features of CRI*.

The shortcomings of the CPC are obvious. CPC calculates the correlation between RR interval and respiratory signal, with the shape of multiple peaks. Physiologically, RSA, the strength of respiration modulation of heart rate should appear as CRF, cannot be multiple peaks as CPC. Through the study of CRI and Cardiopulmonary Coupling (CPC) in distinguishing deep sleep stage, we got the conclusion that CRI does capture physiologically meaningful characteristics of RSA, therefore, well reflect autonomic status in sleep stages. CRI represents the degree of cardiopulmonary resonance, and reflects parasympathetic nerve activity level well.

## Conclusion

Respiratory sinus arrhythmia (RSA) represents a physiological phenomenon of cardiopulmonary interaction. It is known as a measure of efficiency of the circulation system, and a biomarker of cardiac vagal and well-being. In this article, we model RSA as modulation of heart rate by respiration in an interactive cardiopulmonary system with the most effective system state of resonance. Mathematically, it is described by bivariate autoregressive model of respiration series and RR intervals, and quantitatively it is assessed by Granger causality function. The whole model is referred to as Cardiopulmonary Resonance Model (CRM). This method has significant physiological significance in the frequency domain and is convenient for us to explain the experimental results. We suggest using this approach as a universal prepossessing technique which allows a researcher to concentrate on particular properties of the HRV data. Then based on the cardiopulmonary resonance concept, and Granger causality function which is referred to as cardiopulmonary resonance function (CRF) here after, a set of quantitative measures for RSA is proposed, and referred to as Cardiopulmonary Resonance Indices (CRI).

To show the effectiveness of CRM and CRI, two application scenarios, paced breathing and sleep stage discrimination, are studied. It is shown that CRF and CRI provide ideal visual interpretation and numerical measures for cardiopulmonary interactions toward resonance in paced breathing scenario as the paced breathing rate coming down to biofeedback status, and as the sleep stage moves to deep sleep. We draw the conclusion that CRI well represents the degree of cardiopulmonary resonance, and reflects parasympathetic nerve activity level. We think it's a good explanation of the physiological function of RSA and it is also good way to quantify the well-being of human body.

This study has certain limitations. As a measure of RSA under static conditions, CRI was not compared with sympathetic and parasympathetic activity indexes obtained by tilt experiment, nor was it tested under different pathological conditions. We plan to carry out relevant research in the future. In addition, we plan to explore the clinical significance of CRQ and the effects of other physiological activities on heart rate based on NR-HRV data in time and frequency domain. We will gradually accurately analyze the regulatory effects of the autonomic nervous system on various physiological organs and activities through the regulation activities of the autonomic nervous system. It is of great significance for us to understand and monitor the regulation process of the autonomic nervous system in different physiological states.

## Data Availability Statement

All datasets generated for this study are included in the article/supplementary material.

## Author Contributions

JC, JW, and ZH designed the study and performed the research. JC analyzed the data and wrote the paper. ZH and JW reviewed and edited the manuscript. HJ designed and assisted the experiment. All authors read and approved the manuscript.

## Conflict of Interest

The authors declare that the research was conducted in the absence of any commercial or financial relationships that could be construed as a potential conflict of interest.
